# High-quality draft genome sequence of *Gracilimonas tropica* CL-CB462^T^ (DSM 19535^T^), isolated from a *Synechococcus* culture

**DOI:** 10.1186/s40793-015-0088-8

**Published:** 2015-11-11

**Authors:** Dong Han Choi, Chisang Ahn, Gwang Il Jang, Alla Lapidus, James Han, T. B. K. Reddy, Marcel Huntemann, Amrita Pati, Natalia Ivanova, Victor Markowitz, Manfred Rohde, Brian Tindall, Markus Göker, Tanja Woyke, Hans-Peter Klenk, Nikos C Kyrpides, Byung Cheol Cho

**Affiliations:** Biological Oceanography & Marine Biology Division, Korea Institute of Ocean Science and Technology, Ansan, 426-744 Republic of Korea; Microbial Oceanography Laboratory, School of Earth and Environmental Sciences, and Research Institute of Oceanography, Seoul National University, Gwanak-ro, Gwanak-gu Seoul, 151-742 Republic of Korea; Theodosius Dobzhansky Center for Genome Bioinformatics, St. Petersburg State University, St. Petersburg, Russia; Algorithmic Biology Lab, St. Petersburg Academic University, St. Petersburg, Russia; Department of Energy Joint Genome Institute, Genome Biology Program, Walnut Creek, CA USA; Biological Data Management and Technology Center, Lawrence Berkeley National Laboratory, Berkeley, CA USA; Central Facility for Microscopy, HZI – Helmholtz Centre for Infection Research, Braunschweig, Germany; Leibniz Institute DSMZ – German Collection of Microorganisms and Cell Cultures, Braunschweig, Germany; School of Biology, Newcastle University, Newcastle upon Tyne, UK; Department of Biological Sciences, Faculty of Science, King Abdulaziz University, Jeddah, Saudi Arabia

**Keywords:** Genome, *Gracilimonas tropica*, Marine, *Sphingobacteriia*, GEBA

## Abstract

*Gracilimonas tropica* Choi et al. 2009 is a member of order *Sphingobacteriales*, class *Sphingobacteriia*. Three species of the genus *Gracilimonas* have been isolated from marine seawater or a salt mine and showed extremely halotolerant and mesophilic features, although close relatives are extremely halophilic or thermophilic. The type strain of the type species of *Gracilimonas*, *G. tropica* DSM19535^T^, was isolated from a *Synechococcus* culture which was established from the tropical sea-surface water of the Pacific Ocean. The genome of the strain DSM19535^T^ was sequenced through the Genomic Encyclopedia of Type Strains, Phase I: the one thousand microbial genomes project. Here, we describe the genomic features of the strain. The 3,831,242 bp long draft genome consists of 48 contigs with 3373 protein-coding and 53 RNA genes. The strain seems to adapt to phosphate limitation and requires amino acids from external environment. In addition, genomic analyses and pasteurization experiment suggested that *G. tropica* DSM19535^T^ did not form spore.

## Introduction

**Fig. 1:**
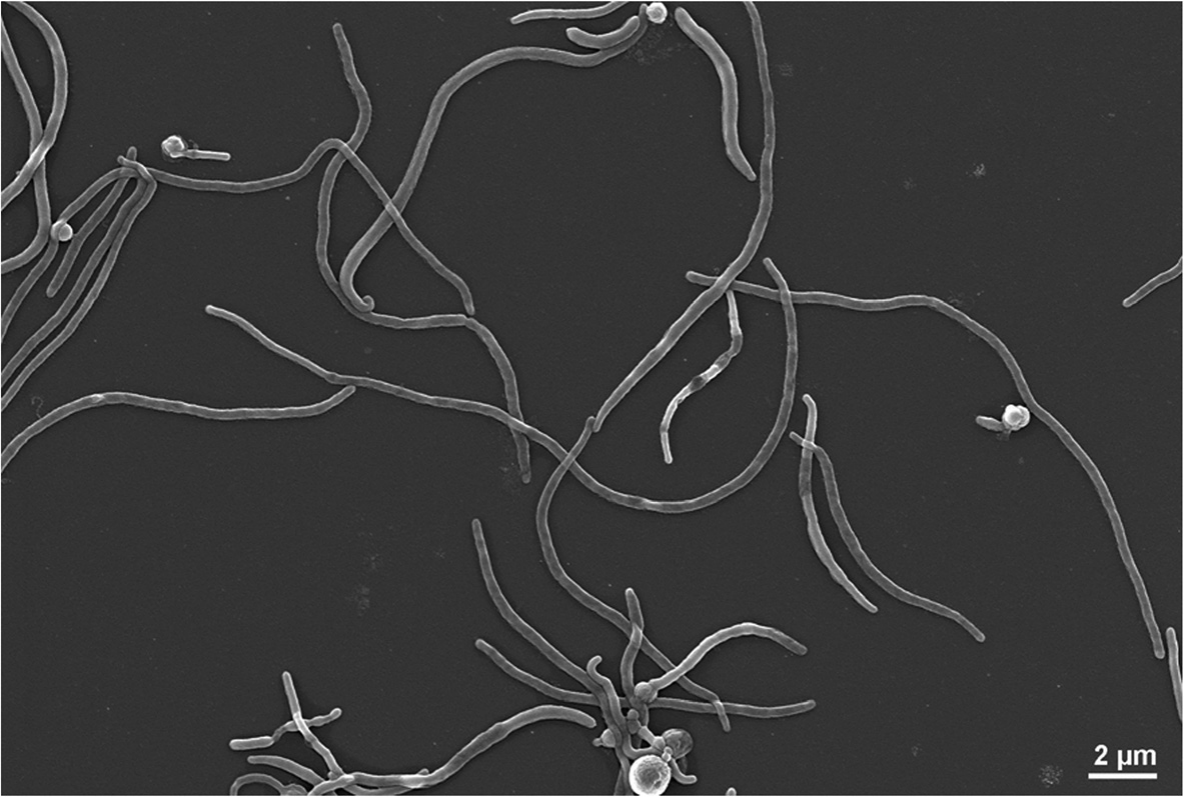
Scanning electron microscopy image of *Gracilimonas tropica* DSM19535^T^

The genus *Gracilimonas* was first established in 2009 [1], and at the time of writing this paper there are three species that comprise this genus, *G. tropica* [1]*,**G. mengyeensis* [2]*,* and *G. rosea* [3]. They are Gram-negative, catalase- and oxidase-positive, aerobic and facultatively anaerobic and have rod-shaped cells (Fig. 1) [1–3]. In addition, they form endospores except *G. mengyeensis* [3]. *Gracilimonas tropica* CL-CB462^T^ (=KCCM 90063^T^ = DSM 19535^T^), the type strain of the type species of the genus *Gracilimonas**,* was isolated from a *Synechococcus* culture which was established from the tropical sea-surface water of the Pacific Ocean [1]. Interestingly, the genus *Gracilimonas* formed a robust clade together with extremely halophilic or thermophilic bacteria (*Salinibacter ruber* and *Rhodothermus marinus*, respectively). On the contrary, *Gracilimonas* species show only extremely halotolerant and mesophilic features. Considering the phenotypic diversity within the clade, their comparative genomic analyses could provide a good clue to understand bacterial adaptation to extreme environments based on genomic context. Here we present a summary of the genomic features of *G. tropica*DSM 19535^T^, which is the first genome-sequenced type strain from the genus *Gracilimonas*.

## Organism information

### Classification and features

**Fig. 2:**
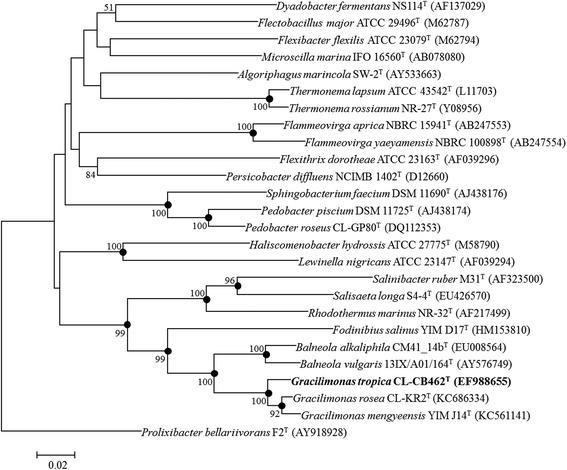
Neighbour-joining tree, based on 16S rRNA gene sequences, showing the phylogenetic position of *G. tropica* DSM 19535^T^. Bootstrap percentages >50 % (based on 1000 resampling) are shown at branching points. Solid circles indicate that the corresponding nodes are also recovered in the maximum-likelihood and maximum-parsimony trees. *Prolixibacter bellariivorans* F2^T^ was used as an outgroup. Bar, 0.02 nucleotide substitutions per site

Phylogenetic analysis based on 16S rRNA gene sequence comparison revealed *G. tropica*DSM19535^T^ is classified into the genus *Gracilimonas* (Fig. 2). The type strains which were most closely related to strain DSM19535^T^ were *Gracilimonas mengyeensis* YIM J14^T^ with 16S rRNA sequence similarity of 96.9 %, and *Gracilimonas rosea* CL-KR2^T^ with a similarity of 96.1 %. Strain DSM19535^T^ is tolerant of high salinity (up to 20 %) with a growth occurring over the range of salinity of 1–20 % (w/v) sea salts (optimum 3–6 %) (Table 1). Growth occurs under either aerobic or facultatively anaerobic conditions. The optimum pH is 7.0–8.0 with a growth range of pH 6–10 (Table 1). The strain was auxotroph for isoleucine and methionine (Table 1). Despite the phylum *Bacteroidetes* is known to be as a non-spore forming group [4], the strain was reported to form endospores, together with *G. rosea* [3]. However, strain DSM19535^T^ could not be asserted to form spore by the genomic analysis (see ‘Insights from the genome sequence’).

**Table 1:** Classification and general features of *G. tropica* DSM 19535^T^ [38, 39]

MIGS ID	Property	Term	Evidence code^a^
	Current classification	Domain *Bacteria*	TAS [40]
		Phylum *Bacteriodetes*	TAS [4]
		Class *Sphingobacteriia*	TAS [41]
		Order *Sphingobacteriales*	TAS [41]
		Genus *Gracilimonas*	TAS [1]
		Species *Gracilimonas tropica*	TAS [1]
		Type strain CL-CB462^T^	TAS [1]
	Gram stain	Negative	TAS [1]
	Cell shape	Rod-shaped	TAS [1]
	Motility	Non-motile	TAS [1]
	Sporulation	Non-sporulation	IDA
	Temperature range	20–40 °C	TAS [1]
	Optimum temperature	35 °C	TAS [1]
	Energy source	Heterotroph	TAS [1]
	Auxotroph for	L-isoleucine, L-methionine	IDA
	Carbon source	Glucose, fructose, aspartate	TAS [1]
MIGS-6	Habitat	Marine, aquatic	TAS [1]
MIGS-6.2	pH	6–10	TAS [1]
MIGS-6.3	Salinity	1–20 % (optimum: 3–6 %)	TAS [1]
MIGS-22	Oxygen requirement	Facultative	TAS [1]
MIGS-15	Biotic relationship	Free living	TAS [1]
MIGS-14	Pathogenicity	Unknown	NAS
MIGS-4	Geographic location	Tropical NW Pacific	TAS [1]
MIGS-5	Sample collection	2009	TAS [1]
MIGS-4.1	Latitude	Unknown	NAS
MIGS-4.2	Longitude	Unknown	NAS
MIGS-4.4	Altitude	0 m	NAS

^a^ Evidence codes - *IDA* Inferred from Direct Assay, *TAS* Traceable Author Statement (i.e., a direct report exists in the literature), *NAS* Non-traceable Author Statement (i.e., not directly observed for the living, isolated sample, but based on a generally accepted property for the species, or anecdotal evidence). These evidence codes are from the Gene Ontology project [42]

By phylogenetic analyses (Fig. 2), the genus *Gracilimonas* formed a sister clade with the genus *Balneola* which shows mesophilic features [5, 6]. At an outer branch, the clade with *Gracilimonas* and *Balneola* formed a robust clade with the moderate halophilic *Fodinibius salinus* (Fig. 2). Moreover, at a deeper branch, the clade formed a robust association with a clade that includes the thermophilic genus *Rhodothermus* [7] and the genus of extremely halophilic *Salinibacter* [8], despite the relatively low (ca. 80 %) similarities between the two clades. Thus, the phylogentically robust clade contains both extremophiles and non-extremophiles.

Auxotrophy for amino acids was examined using a minimal medium (glucose, 2 g; pyruvate, 0.3 g; K_2_HPO_4_, 3 g; NaH_2_PO_4_, 1 g; NH_4_Cl, 1 g; MgSO_4_•7H_2_O, 0.3 g; 1 ml of Holden’s trace elements [9]; 1 ml of Balch’s vitamin solution [10]; 1 L of artificial seawater [11]) supplemented with 0.3 mM or 3 mM of all amino acids except a focal amino acid. The strain could not grow in minimal medium without supplementation of L-isoleucine and L-methionine. But, the strain did not require other amino acids (L-alanine, L-arginine, L-asparagine, L-aspartate, L-cysteine, L-glutamate, L-glutamine, glycine, L-histidine, L-lysine, L-phenylalanine, L-proline, L-serine, L-threonine, L-tryptophan, L-tyrosine and selenocysteine) for growth.

## Genome sequencing information

### Genome project history

A culture of DSM 19535^T^ (strain CL-CB462^T^) was selected for sequencing on the basis of its phylogenetic position [12, 13], and is part of the Genomic Encyclopedia of Type Strains, Phase I: the one thousand microbial genomes project [14], a follow-up of the Genomic Encyclopedia of *Bacteria* and *Archaea* pilot project [15], which aims in increasing the sequencing coverage of key reference microbial genomes and to generate a large genomic basis for the discovery of genes encoding novel enzymes [16]. The one thousand microbial genomes-I is the first of the production phases of the Genomic Encyclopedia of *Bacteria* and *Archaea*: sequencing a myriad of type strains initiative [17] and a Genomic Standards Consortium project [18]. The genome project is deposited in the Genomes On Line Database [19] and the genome sequence is available from GenBank. Sequencing, finishing and annotation were performed by the DOE Joint Genome Institute (JGI) using state of the art sequencing technology [20]. A summary of the project information is presented in Table 2.

**Table 2:** Project information

MIGS ID	Property	Term
MIGS-31	Finishing quality	Level 2: High Quality Draft
MIGS-28	Libraries used	Illumina Std shotgun library
MIGS-29	Sequencing platforms	Illumina
MIGS-31.2	Sequencing coverage	421×
MIGS-30	Assemblers	Velvet v. 1.1.04, ALLPATHS v. R41043
MIGS-32	Gene calling method	Prodigal v2.5
	NCBI project ID	169,820
	Genbank ID	AQXG00000000
	Genbank Date of Release	December 12, 2013
	GOLD ID	Gp0013655
	BIOPROJECT	PRJNA169820
MIGS-13	Source Material Identifier	DSM 19,535
	Project relevance	GEBA-KMG, Tree of Life

### Growth conditions and genomic DNA preparation

*G. tropica*DSM 19535^T^, was grown in DSMZ medium 514 (Bacto Marine Broth) [21] at 28 °C. Genomic DNA was isolated from about 0.5 g of cell paste using Jetflex Purification Kit (Epicentre MGP04100) following the standard protocol as recommended by the manufacturer with an additional protease K (50 μl; 21 mg/ml) digest for 60 min. at 58 °C followed by addition of 200 μl Protein Precipitation Buffer after protein precipitation and overnight incubation on ice [22]. DNA was quality controlled according to JGI guidelines and is available through the DNA Bank Network [23].

### Genome sequencing and assembly

The draft genome was generated using Illumina technology [24]. An Illumina Std shotgun library was constructed and sequenced using the Illumina HiSeq 2000 platform which generated 14,058,618 reads totaling 2108.8 Mbp. All general aspects of library construction and sequencing performed at the JGI can be found at [25]. All raw Illumina sequence data was passed through DUK, a filtering program developed at JGI, which removes known Illumina sequencing and library preparation artifacts (Mingkun L, Copeland A, Han J. DUK, unpublished, 2011). Artifact filtered sequence data was then screened and trimmed according to the k–mers present in the dataset (Mingkun L. kmernorm, unpublished, 2011). High–depth k–mers, presumably derived from MDA amplification bias, cause problems in the assembly, especially if the k–mer depth varies in orders of magnitude for different regions of the genome. Reads with high k–mer coverage (>30 × average k–mer depth) were normalized to an average depth of 30×. Reads with an average kmer depth of less than 2× were removed. Following steps were then performed for assembly: (1) normalized Illumina reads were assembled using Velvet version 1.1.04 [26], (2) 1–3 Kbp simulated paired end reads were created from Velvet contigs using wgsim [27], (3) normalized Illumina reads were assembled with simulated read pairs using Allpaths–LG (version r41043) [28]. Parameters for assembly steps were: 1) Velvet (velveth: 63 –shortPaired and velvetg: −very clean yes –exportFiltered yes –min contig lgth 500 –scaffolding no –cov cutoff 10), 2) wgsim (−e 0 –1 100 –2 100 –r 0 –R 0 –X 0), 3) Allpaths–LG (PrepareAllpathsInputs: PHRED 64 = 1 PLOIDY = 1 FRAG COVERAGE = 125 JUMP COVERAGE = 25 LONG JUMP COV = 50, RunAllpathsLG: THREADS = 8 RUN = std shredpairs TARGETS = standard VAPI WARN ONLY = True OVERWRITE = True). The final draft assembly contained 48 contigs in 48 scaffolds. The total size of the genome is 3.8 Mbp and the final assembly is based on 457.7 Mbp of Illumina data. Based on a presumed genome size of 5Mbp, the average coverage of the genome was 421 × .

### Genome annotation

Genes were identified using Prodigal [29] as part of the DOE-JGI Annotation pipeline [30, 31] followed by a round of manual curation using the JGI GenePRIMP pipeline [32]. The predicted CDSs were translated and used to search the National Center for Biotechnology Information non-redundant database, UniProt, TIGRFam, Pfam, PRIAM, KEGG, COG, and InterPro databases. Additional gene prediction analysis and functional annotation was performed within the Integrated Microbial Genomes [33].

## Genome properties

The genome of the strain is 3,831,242 bp long and comprises 48 contigs ranging 1177 to 783,752 bp, with an overall GC content of 42.9 % (Table 3). Of the 3426 genes predicted, 3373 were protein coding genes, and 53 were RNA genes. A total of 2413 genes (70.4 %) were assigned a putative function while the remaining ones were annotated as hypothetical or unknown proteins. The distribution of genes into COG functional categories is presented in Table 4. The properties and the statistics of the genome are summarized in Tables 3 and 4.

**Table 3:** Genome statistics

Attribute	Number	% of Total^a^
Genome size (bp)	3,831,242	100.00
DNA coding (bp)	3,482,093	90.89
DNA G + C (bp)	1,645,319	42.94
DNA scaffolds	48	100.00
Total genes	3426	100.00
Protein-coding genes	3373	98.45
RNA genes	53	1.55
Pseudo genes	0	
Genes in internal clusters	1042	30.41
Genes with function prediction	2413	70.43
Genes assigned to COGs	1931	56.36
Genes with Pfam domains	2557	74.64
Genes with signal peptides	385	11.24
Genes with transmembrane helices	912	26.62
CRISPR repeats	0	

^a^The total is based on either the size of the genome in base pairs or the total number of protein coding genes in the annotated genome

**Table 4:** Number of genes associated with general COG functional categories

Code	Value	% age	Description
J	138	6.56	Translation, ribosomal structure and biogenesis
A	0	0.00	RNA processing and modification
K	112	5.32	Transcription
L	107	5.09	Replication, recombination and repair
B	1	0.05	Chromatin structure and dynamics
D	24	1.14	Cell cycle control, cell division, chromosome partitioning
V	53	2.52	Defense mechanisms
T	96	4.56	Signal transduction mechanisms
M	175	8.32	Cell wall/membrane biogenesis
N	17	0.81	Cell motility
U	44	2.09	Intracellular trafficking and secretion
O	93	4.42	Posttranslational modification, protein turnover, chaperones
C	133	6.32	Energy production and conversion
G	95	4.52	Carbohydrate transport and metabolism
E	178	8.46	Amino acid transport and metabolism
F	60	2.85	Nucleotide transport and metabolism
H	96	4.56	Coenzyme transport and metabolism
I	80	3.80	Lipid transport and metabolism
P	103	4.90	Inorganic ion transport and metabolism
Q	53	2.52	Secondary metabolites biosynthesis, transport and catabolism
R	263	12.50	General function prediction only
S	183	8.79	Function unknown
-	1495	43.64	Not in COGs

The total is based on total number of protein coding genes in the annotated genome

## Insights from the genome sequence

Based on genomic analysis of the metabolic features, *G. tropica*DSM19535^T^ is predicted to be an auxotroph for L-lysine, L-phenylalanine, L-tyrosine, L-arginine, L-aspartic acid, L-isoleucine, L-proline, and L-methionine. In the auxotroph test, however, the strain was found to be auxotroph only for L-isoleucine and L-methionine (Table 1). This discrepancy might be due to missing annotations of essential genes by incomplete sequencing or presence of unknown genes related with transport and/or assimilation. In addition, despite selenocysteine was one of essential amino acids required for growth by the genomic analysis, the strain could grow in a medium without selenocysteine. Genome analysis also revealed that strain DSM19535^T^ has a copper-containing nitrite reductase gene (*nirK*) homolog, suggesting that the strain may transform nitrite to nitric oxide (NO) under low oxygen or anoxic conditions. In addition, the strain contains *DnrN* (nitric oxide-dependent regulator) gene and this may protect cells from nitrosative stress [34]. However, the nitrate, nitric oxide and nitrous oxide reductases involved in denitrification were not found. The strain has an ATP-dependent glutamine synthetase and a NADPH-dependent glutamate-oxoglutarate amidotransferase, and thus can assimilate ammonia into glutamate and glutamine. In the strain, ammonium may be transported by an ammonium transport protein. Genes participating in phosphate metabolism were also identified in the genome of the strain DSM19535^T^. Inorganic pyrophosphatase catalyzing the conversion of pyrophosphate to phosphate ion, and polyphosphate kinase catalyzing the formation of polyphosphate from ATP were found in the genome. The strain has several genes of Pho regulon (*phoH*, *phoU*, *phoR* and *phoB*) mediating an adaptive response to inorganic phosphate limitation but not high affinity phosphate binding protein and transporter (*pstS* and *pstACB*). In addition, the strain may hydrolyze phosphate groups from many types of organic molecules using alkaline phosphatase.

In the previous study, *G. tropica*DSM19535^T^ was reported to be able to form spores [1]. The spore-formation is very unusual in the phylum *Bacteroidetes* [4]. Despite four and five proteins were annotated as stage II sporulation protein E (SpoIIE) and sporulation related domain, respectively, by search against the Pfam database, more than a hundred sporulation-related genes identified in *Bacillus subtilis* 168^T^ were absent from the genome of strain DSM19535^T^. Further, the genes found in *G. tropica* were also found in genomes of phylogenetically close but non-sporulating genera, *Balneola vulgaris*DSM 17,893 and *Salisaeta longa*DSM2114. Therefore, further tests to examine spore-formation were conducted in this study. Consistent with the previous study, spore-like spherical cells were found after malachite green staining. However, after pasteurization at 60 °C for 10 and 20 min and 80, 90 and 100 °C for 10 min, re-growth of cells was never observed, suggesting that the coccoid cells may not be endospore. Actually, non-spore but spore-like spherical cells were also found in aging cultures of a variety of non-sporulating bacteria including *Salinispira pacifica* belonging to the phylum *Spirochaetae*, *Prolinoborus fasciculus* belonging to the class *Betaproteobacteria* and *Anaerophaga thermohalophila* belonging to the phylum *Bacteroidetes* [35–37]. The genomic analyses and pasteurization experiment convincingly suggested that the spore-like coccoid cells of *G. tropica*DSM19535^T^ are not endospores.

## Conclusion

The genome of a member belonging to the genus *Gracilimonas* in the phylum *Bacteroidetes* is reported here. In addition to detailed information of genome sequencing and annotation, genetic characteristics related with nitrogen and phosphorus utilization could be understood on the basis of genomic analyses. In addition, genomic analyses and pasteurization experiments suggested that *G. tropica*DSM19535^T^ does not form spores.
